# Supplementary figures and images for: Older Women Who Practiced Physical Exercises before the COVID-19 Pandemic Present Metabolic Alterations and Worsened Functional Physical Capacity after One Year of Social Isolation
Source: Healthcare (Basel). 2022 Sep 9;10(9):1736. doi: 10.3390/healthcare10091736 (PMC9498301; doi:10.3390/healthcare10091736)

## Timed get up and go test (TUGT)

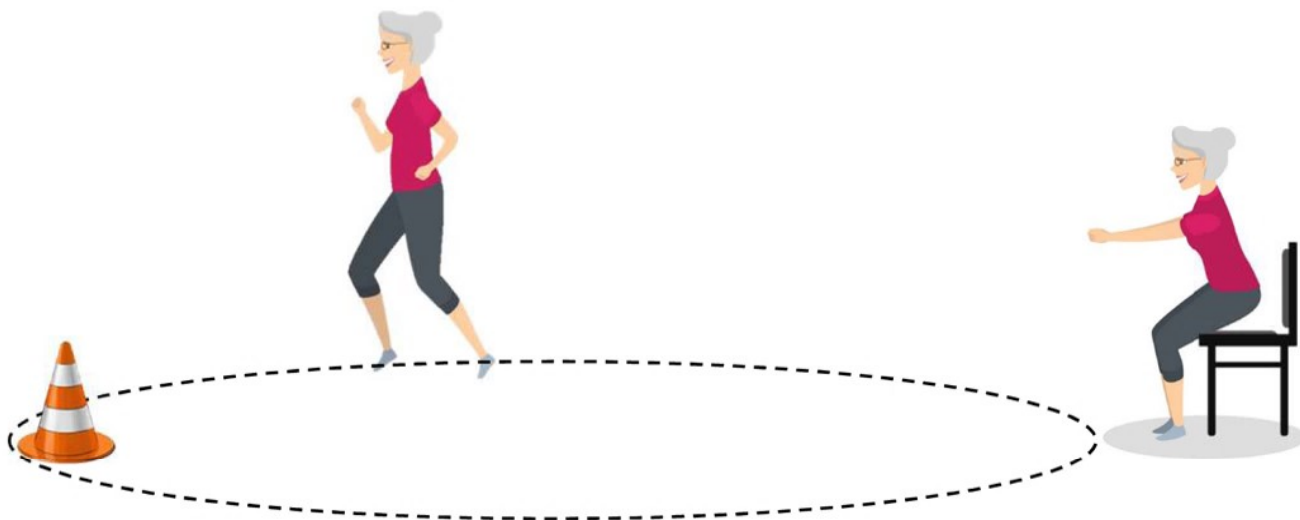

## Handgrip

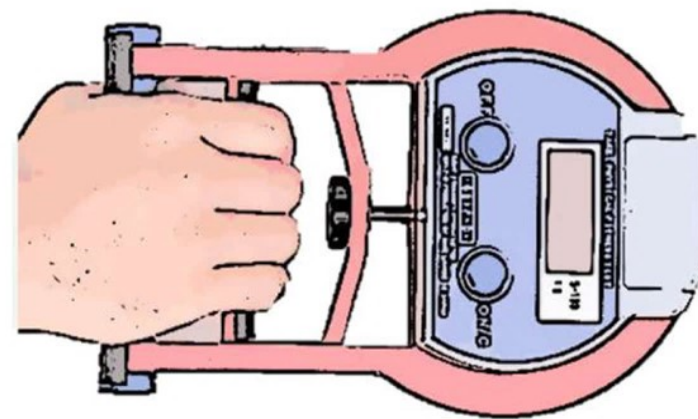

## Gait speed (GS)

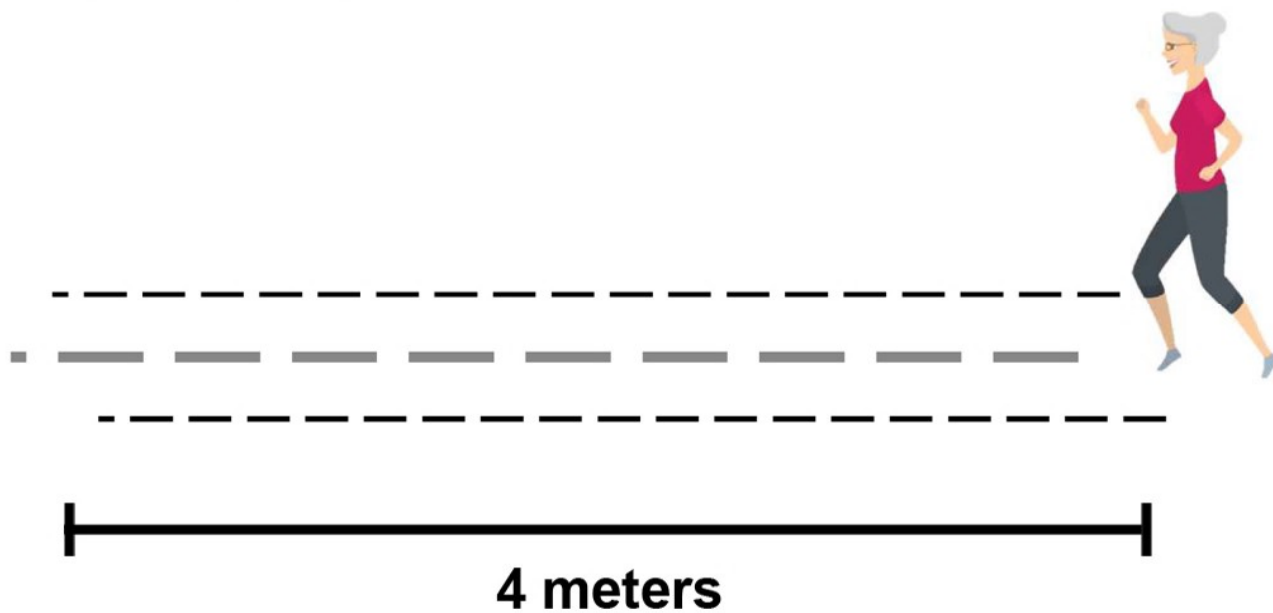

Supplement: Supplementary file 1 [file healthcare-10-01736-s001.zip › healthcare-1869204-supplementary.pdf]
